# Segregated brain state during hypnosis

**DOI:** 10.1093/nc/niab002

**Published:** 2021-03-10

**Authors:** Jarno Tuominen, Sakari Kallio, Valtteri Kaasinen, Henry Railo

**Affiliations:** Department of Psychology and Speech-Language Pathology, Assistentinkatu 7, 20014 University of Turku, Turku, Finland; Turku Brain and Mind Center, 20014 University of Turku, Turku, Finland; Department of Psychology and Speech-Language Pathology, Assistentinkatu 7, 20014 University of Turku, Turku, Finland; School of Biosciences, Högskolevägen 3, 54128 University of Skövde, Skövde, Sweden; Turku Brain and Mind Center, 20014 University of Turku, Turku, Finland; Division of Clinical Neurosciences, 20014 University of Turku, Turku, Finland; Turku University Hospital, Kiinamyllynkatu 4-8, 20521 Turku, Finland; Department of Psychology and Speech-Language Pathology, Assistentinkatu 7, 20014 University of Turku, Turku, Finland; Turku Brain and Mind Center, 20014 University of Turku, Turku, Finland; Turku University Hospital, Kiinamyllynkatu 4-8, 20521 Turku, Finland; Department of Clinical Neurophysiology, University of Turku, P.O. Box 52, 20521 Turku, Finland

**Keywords:** brain dynamics, hypnosis, metastability, transcranial magnetic stimulation

## Abstract

Can the brain be shifted into a different state using a simple social cue, as tests on highly hypnotizable subjects would suggest? Demonstrating an altered global brain state is difficult. Brain activation varies greatly during wakefulness and can be voluntarily influenced. We measured the complexity of electrophysiological response to transcranial magnetic stimulation in one ‘hypnotic virtuoso’. Such a measure produces a response arguably outside the subject’s voluntary control and has been proven adequate for discriminating conscious from unconscious brain states. We show that a single-word hypnotic induction robustly shifted global neural connectivity into a state where activity remained sustained but failed to ignite strong, coherent activity in frontoparietal cortices. Changes in perturbational complexity indicate a similar move towards a more segregated state. We interpret these findings to suggest a shift in the underlying state of the brain, likely moderating subsequent hypnotic responding.

## Introduction

Hypnotic suggestions can have profound effects on behaviour and experience in some individuals, e.g. producing realistic-seeming hallucinations (Oakley and Halligan 2013). At the heart of this debate is the question whether hypnosis changes the way the individual processes information. While this question is often framed by contrasting so-called state theories of hypnosis with socio-cognitive (‘non-state’) theories, this dichotomy is simplified, and hypnosis is likely best be explained by a combination of the two views (Kirsch and Lynn 1995; Kallio and Revonsuo 2003). Here, our aim is not to test between these two classes of theories. Instead, we solely focus on characterizing the global neural-level changes that accompany hypnosis. Our specific aim is to test whether the brain can be shifted to a fundamentally different dynamic state based on hypnotic induction.

### Neural basis of hypnosis

What kinds of changes in brain activation and neural dynamics accompany hypnosis? While numerous studies (e.g. Kosslyn *et al.* 2000; Fingelkurts *et al.* 2007; Jiang *et al.* 2017) have shown hypnosis to be associated with changes in neural activation patterns, these findings are both inconclusive and inconsistent (Landry and Raz 2017). The observed changes could also simply reflect normal variation in brain activity (see e.g. Mazzoni *et al.* 2013). Furthermore, most studies have included a specific suggestion which makes it difficult to distinguish between changes in the background state configuration and the task-specific changes engaged by the suggestion. In this study, we concentrate on ‘neutral hypnosis’*—*a hypnotic induction without further suggestions—to investigate the changes brought about in brain dynamics by a simple single-word post-hypnotic induction.

Three networks have currently been implicated within hypnosis research: the salience network (SN), the central executive network (CEN), and the Default Mode Network (DMN) (Landry and Raz 2017). The SN and the CEN are essential for higher-order cognition, the former with awareness of internal and external events, and the latter with attention and anticipation, whereas the DMN (e.g. Gusnard and Raichle 2001; Raichle 2015) is concerned with internal attention, task-unrelated thought, self-referencing, and social cognition. In their meta-analytic review, Landry and Raz (2017) assessed the activity and connectivity between these three networks as likely enablers of the absorption that facilitates top-down effects and hypnotic responding. Interestingly they found no consistent support for any of the networks, only for the lingual gyrus—a brain area associated with mental imagery. It can be concluded that while hypnotic induction alters the background dynamics of the brain to mediate the effects of specific suggestions (e.g. Oakley 2012, 379), there is little consensus within the extant data for its sufficient causes.

### What is the perturbational complexity index?

Reduced states of consciousness—including vegetative state, minimally conscious state, anaesthetized state, coma, and NREM sleep—can all be distinguished from wakeful consciousness by using transcranial magnetic stimulation (TMS) and quantifying the resulting patterns of activation (Casali *et al.* 2013; Sarasso *et al.* 2015; Casarotto *et al.* 2016). This ‘perturbational complexity’ index (PCI) allows quantification of the brain’s potential both to segregate (i.e. differentiate) and integrate information (Casali *et al.* 2013). In practice, it is a measure of normalized algorithmic complexity arrived at by compressing the spatiotemporal pattern of perturbed cortical activations. Thus, a PCI value of zero indicates a completely integrated system, and a value of one a completely segregated or random system. Studies have shown that patients with reduced states of consciousness have a lower PCI, with values ˂0.31 generally considered to indicate unconsciousness (Casali *et al.* 2013; Sarasso *et al.* 2015; Casarotto *et al.* 2016). Normal wakeful consciousness is associated with intermediate PCI values, reflecting an optimal balance between integration and segregation (Casali *et al.* 2013; Casarotto *et al.* 2016). ^1^ As of now there is no indication that PCI can be brought under volitional control, and a not-yet reviewed preprint indicates it to have a high intra- and inter-subject reliability (Caulfield *et al.* 2020).

PCI is often motivated through the Integrated Information Theory (IIT) of consciousness (Oizumi *et al.* 2014), which states that consciousness corresponds to different brain areas forming a complex system. PCI is used as a proxy of information integration, because it allows to probe the complexity of the system’s response to perturbation (Casali *et al.* 2013; Sarasso *et al.* 2015; Casarotto *et al.* 2016). PCI is also intimately linked with the concept of metastability which, just like IIT, denotes systems states where information integration and differentiation coexist in an optimal fashion. In metastable state, brain areas can share information while the overall system maintains the ability to change configuration dynamically: brain areas can engage with and disengage from the network while maintaining functional specificity (Kelso 2012; Tognoli and Kelso 2014). This is presumed (Kelso 2012) to allow adaptive behaviour through coordination of external (sensory) and internal (e.g. task-set) stimuli. To demonstrate the link between metastability and IIT, consider Mediano *et al.* (2016), who modelled metastable systems using Kuramoto oscillators. Metrics denoting metastability, information integration, and entropy all peaked around the same ‘transition region where the oscillators operate in a critical regime poised between order and disorder’ (Mediano *et al.* 2016, 2). Shift away from this region mediating complex behaviours is a shift towards less complex, integrated, or differentiated state (or hypersynchronized vs. desynchronized oscillatory states, respectively). We propose that this type of dynamic system’s view may shed important new information into how brain activity in hypnosis differs from normal wakefulness. A recent study of TMS-evoked activation in a single highly-trained subject undergoing self-induced cognitive trance found differences in, e.g. phase-locking, cortical synchrony, and increased phenomenological dissociation of the environment (Gosseries *et al.* 2019).

### Current study

An arguably fruitful way to understand hypnosis is provided by studying the so-called ‘hypnotic virtuosos’; participants who are very highly hypnotizable and can respond a broad spectrum of hypnotic suggestions (Orne 1971; Weitzenhoffer 2000; Kallio and Revonsuo 2003). They provide a good starting point for studying the neural basis of hypnosis, exemplifying a token example of what generally is considered hypnosis in its clearest form. This approach should be appended by larger study designs from throughout the spectrum of hypnotic responding using the same paradigms. This would allow us to begin work on understanding both the individual differences and share commonalities of neural mechanisms of hypnotic responding.

Such a two-tier approach is in-line with the majority of recommendations of a recent review, staking the path for a better understanding of hypnosis (Jensen *et al.* 2017). The only note of discord between these suggestions and our current study is that we opted to use within participant control ‘condition’, not a control ‘group’. The motivation for this is 2-fold: given the high inter-individual variation in hypnosis research (Landry and Raz 2017) we wish to test our tentative hypothesis on a person who can consistently and reliably produce a strong hypnotic response, including hallucinatory experiences and resultant changes in behavioural measures (Kallio *et al.* 1999; Fingelkurts *et al.* 2007; Kallio *et al.* 2011; Kallio and Koivisto 2016). Second, following the single-subject study design tradition, we avoid generalizable claims and opt to isolate the topic of interest in this one atypically hypnotizable participant: given hypnosis is a true phenomenon with high variability, this showcases one possible instantiation of such a mechanism. By providing one well-controlled study on the exact changes in brain state configuration we can note this to be one way to undergo hypnosis, and make the argument that this in itself is a useful and fruitful approach to forward future research considerations. By simplifying our research setting to minimal complexity we minimize the presence of external factors that could affect our chosen measurements beyond hypnosis.

For our study, we opted for a perturbational TMS–EEG approach because of the way it allows characterization of the brain’s functional state, while the outcome (spread of TMS-elicited activation) is considered to rest beyond the subject’s voluntary control. We hypothesized that the psychological and behavioural changes associated with hypnosis are produced by a brain state in which the segregation/integration balance is different from normal waking consciousness: specifically, it is possible PCI should decrease as the subject enters hypnosis, reminiscent of other states where consciousness begins to fade (Casali *et al.* 2013) and brain activation becomes less complex. Such a prediction seemed sensible, given the relaxation techniques through which hypnotic induction is usually performed and the phenomenological relaxed experience often reported to accompany hypnosis (Banyai and Hilgard 1976; Rainville *et al.* 2002; Kahn and Hobson 2003; Wark 2006). It should be noted, however, that while experiential similarities have been cited, sleep and hypnosis are not typically considered to be similar to each other neurobiologically (Evans 1979). Meanwhile, any increase in PCI under hypnosis would indicate increased segregation in the thalamocortical networks. Thus, either a decrease or an increase in PCI would indicate a shift from metastable to less optimal dynamic state. By contrast, if hypnosis lies within the continuum of normal wakeful consciousness, PCI under hypnosis should not change from relaxed normal baseline. Additionally, we assessed the spatiotemporal dynamics following the TMS-perturbation to achieve a more detailed analysis of the underlying dynamics.

## Materials and Methods

### Participant

T.S.H. is a highly hypnotizable, 51-year-old female office worker with no history of psychiatric or neurological illness. She gave informed written consent to take part in the study and received a small fee for participation. She has been extensively evaluated in previous studies (Kallio *et al.* 1999; Fingelkurts *et al.* 2007; Kallio *et al.* 2011; Kallio and Koivisto 2016). She rates at maximum in the Stanford suggestibility scale (C, Weitzenhoffer and Hilgard 1962) designed to assess individual suggestibility, and is considered a model example of a somnambulic or ‘hypnotic virtuoso’ individual. She is able to experience a vast variety of hypnotic cognitive phenomena including vivid auditory and visual hallucinations, both positive and negative. These phenomena can also be induced post-hypnotically. The single-word induction allows for a consistent and controllable research setting. This makes her an ideal research participant for a possible mechanism of hypnosis-related changes in brain dynamics. Description of the relevant post-hypnotic suggestions and T.S.H.’s psychometric profile are available in (Kallio *et al.* 2011).

### Procedure and stimuli

Electroencephalography (EEG; 64-electrode, 5 kHz sampling rate, NeurOne Tesla amplifier, Mega Electronics) DC recording was combined with single-pulse TMS. Electrode (Ag/AgCl electrodes) placement followed the international 10–20 system, with a ground electrode placed on the forehead and a reference electrode on the nose. An additional electrode was placed below the right eye and another at the right outer canthus to measure eye movements. To minimize TMS artefacts, impedances were brought to ∼1 kΩ before recording. Single biphasic TMS pulses at 65% maximal output were applied with a NexStim Eximia stimulator to the right calcarine sulcus with current direction lateral to medial during the second pulse phase. The location was chosen because it is easily accessible, feels comfortable to the subject, and was used in a previous study (Railo *et al.* 2016). Note that PCI is not significantly influenced by stimulation location (Casali *et al.* 2013). The stimulation location was neuro-navigated and monitored continuously using the NexStim NBS system, guided by 3T MRI images of the subject’s brain. The subject sat in a relaxed position with eyes closed. A chin rest was used to stabilize head motion. The subject wore foam earplugs through which constant Gaussian white noise was played to attenuate the sound of the TMS pulse. The test session took just over 2 h in total, including setup for the EEG recording. Altogether 167 pulses were applied in the hypnosis condition and 172 in the baseline condition within six alternating blocks. The subject was given time to rest between blocks.

Over the course of the session, the subject was hypnotized and the hypnosis cancelled via single-word cues delivered as post-hypnotic suggestion. This was facilitated by means of a prior hypnotization, using a standard hypnotic induction procedure (Shor and Orne 1962). T.S.H. was instructed that the word ‘hypno’ would function as a cue for hypnosis and ‘base’ for return to normal waking state. A video of the single-word cue induction of T.S.H. has been made available as a supplement to a previous publication (Kallio *et al.* 2011).

A short auditory stimulus was presented on average every 10 s. The subject was instructed to react via a simple button press on a keyboard. The auditory stimulus and button press were introduced to assess possible adverse effects during the stimulation and not included in the analysis. If the subject should fail to respond to the stimulus, the researchers would know to abort the session. Each auditory stimulus was separated by a minimum of 1.2 s from a TMS pulse.

### Ethical considerations

The study was approved by the ethics committee of the Hospital District of Southwest Finland in alignment with the Helsinki declaration. Exclusion criteria for good TMS practices (Rossi *et al.* 2009) were followed. Informed consent was gained before the testing procedure began, and the procedure could be aborted at any time without ramifications.

### Data processing

The EEG data were pre-processed using EEGlab version 13.4.4b (Delorme and Makeig 2004) on Matlab 2014b. To remove the TMS pulse artefact, 5 milliseconds (ms) data were cut and interpolated by a third-order polynomial curve (Reichenbach *et al.* 2011). Further pre-processing consisted of 500 Hz re-sampling, re-referencing to average, 0.1 Hz high-pass filtering, and line-noise removal using CleanLine.^2^ Eye blinks and remaining TMS artefacts were removed using ICA (runica algorithm) and visual inspection. The EEG recording was then segmented into 800 ms epochs, beginning from 400 ms before TMS stimulation. Epochs containing outliers (joint probability activity limit = 4 SDs) or where EEG amplitude crossed a threshold (±175 µV) relative to baseline were removed: 28 in all. Source reconstruction was carried out using SPM8 (Litvak *et al.* 2011), calculating a linear mapping between EEG sensory activity and dipoles distributed over the cortical surface (Dale and Sereno 1993). Montreal Neurological Institute brain-template mesh was used to model cortical anatomy, with the boundary elements model as a forward model. An empirical Bayesian approach (Lopez *et al.* 2014) with minimum norm constraint was used to calculate the inverse reconstruction. A 40 Hz low-pass filter was applied during source reconstruction. The source reconstruction was modelled for single trials as per (Casali *et al.* 2013) to keep the method for calculating PCI identical to the original. After pre-processing, the baseline and hypnosis datasets included 164 and 146 pulses respectively.

A non-parametric ‘bootstrap procedure’ (Casali *et al.* 2013) was run to estimate which dipoles were statistically significantly modulated by TMS. This method determines when and where the reconstructed post-stimulus activation differs in a statistically significant way from pre-stimulus activation. First, each source activity was *z*-transformed. Second, single-trial pre-stimulus samples were randomly shuffled to calculate a surrogate baseline for each source activity. Maximum absolute value across all sources was calculated to correct for multiple comparisons (Pantazis *et al.* 2005). The procedure was repeated 1000 times to obtain a null distribution. One-tailed 0.01 alpha level was used as threshold to determine which sources at a given time point differed in a statistically significant way from baseline. This yields a binary matrix of ones and zeros, denoting whether activity in a given dipole at a given time point is modulated by TMS. The spatiotemporal pattern of significant current density (SCD; Casali *et al.* 2010)—Fig. 2c in the main text—shows the sum of source currents per time point in all sources displaying statistically significant modulation by TMS. Figure 2d shows the standard deviation of individual source activations across trials (the sum of all standard deviations across all dipoles is plotted). Broadband phase-locking (bPL) was calculated from single-trial source activations using the Hilbert transform. bPL values describe similarity of the phase across trials, with a range from zero (indicating random phases) to one (perfect phase-locking) (Sinkkonen *et al.* 1995; Tallon-Baudry *et al.* 1996).

PCI calculation was based on the binary matrix of significant sources. This was done by determining the Lempel-Ziv complexity of the matrix and normalizing the result by the source entropy of the statistically significant source activations (Casali *et al.* 2013): i.e. PCI values range from 0 to 1. Values close to 0 indicate that the EEG response can be characterized with little information; values close to 1 indicate a highly complex response. In other words, the PCI values describe the entropy of the neural response elicited by the TMS pulse. PCI is seen as indicative of the brain’s capacity to integrate information (Storm *et al.* 2017), with intermediate values understood to reflect an optimal balance between integration and segregation. While there is some individual variation in subjects’ PCI scores during waking consciousness, the differences observed between states are robust.

**Figure 1.**
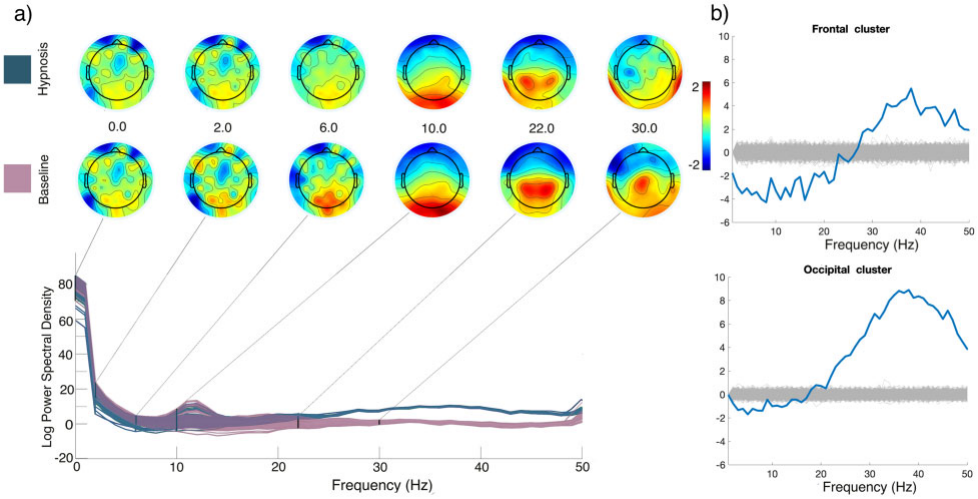
Spectral power differences (**a**) between hypnosis (blue) and baseline (pink) in the pre-pulse EEG data. Topographical plots indicate activation for various frequencies. Frontal and occipital electrode clusters (**b**) compared to a null distribution arrived at by calculating 1000 random permutations, shown individually in light grey lines. Auditory control stimuli and responses were excluded from the analyses.

A permutation procedure was employed on the source-reconstructed single-trial data to assess if the observed differences between baseline and hypnosis conditions were statistically significant. To assess statistically significant differences in SCD, a null distribution was obtained by randomly shuffling condition labels (baseline vs. hypnosis condition) 1000 times. This allowed calculation of a null distribution of differences—per source and time point—expected by chance. Spatiotemporal distribution of statistically significant differences in source currents is shown in Supplementary data, Fig. S1. Statistically significant differences in SCD are found in Fig. 1c.

A similar permutation approach was employed to determine whether the difference in PCI between baseline and hypnosis was statistically significant. This involved shuffling the condition labels, and calculating the binary matrix of source activations and corresponding PCI multiple times, to yield a null distribution to which the observed PCI difference could be compared. This allowed us to assess whether the PCI findings were due to normal individual variation or denoted an actual difference between states. To reduce computational load, source activations were converted to binary form using a procedure (Schartner *et al.* 2015) that takes less time than the bootstrap procedure (Casali *et al.* 2013). The threshold used for statistical significance at a given source was calculated as the mean of the absolute values of the analytic signal, as determined by Hilbert transform (Schartner *et al.* 2015). The PCIs of two surrogate datasets, corresponding to the baseline and hypnosis conditions under the null hypothesis, was then determined. The surrogate datasets were created by randomly shuffling the labels of trials in the original data 1000 times. The observed difference in PCI between surrogate ‘baseline’ and ‘hypnosis’ conditions constitutes the aforementioned null distribution to which the observed difference in PCI between actual baseline and hypnosis conditions can then be compared. This rests on the assumption that PCI, as calculated using the bootstrap procedure (Casali *et al.* 2013) or thresholding procedure (Schartner *et al.* 2015) will be essentially the same. Supplementary data, Fig. S2 shows that this is indeed the case. Additionally, to investigate whether differences in PCI are attributable specifically to perturbation-related effects, or whether they more generally track global features of the state, we ran additional PCI analyses on the pre-pulse time period. Power spectral density was estimated separately for both conditions for each electrode, and differences between the two conditions in frontal and occipital clusters were compared to a null distribution arrived at by shuffling and permuting data from both conditions a 1000 times. For this analysis, the post-pulse activation, the beep, and the corresponding button press response were removed.

The TMS–EEG datasets are available through the Open Science Framework at https://osf.io/e2pkt/.

## Results

**Figure 2.**
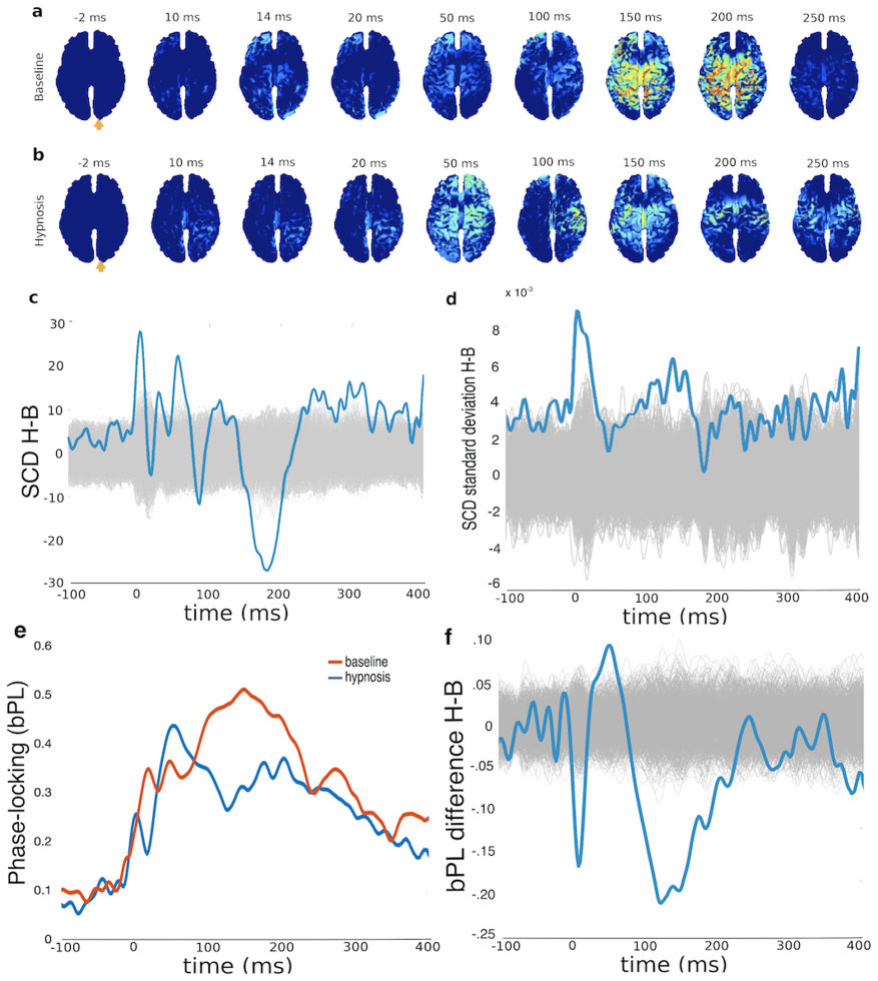
Electrophysiological responses to TMS under hypnosis and (relaxed) baseline state. The spatiotemporal distribution of statistically significant, source-reconstructed. TMS-evoked activation (**a**) under baseline and (**b**) under hypnosis reveal clear differences. An orange arrow indicates the site of TMS stimulation. Panel (**c**) plots the difference in SCD (sum over all dipoles in Panels a and b) between hypnosis and baseline compared to a null distribution. Panel (**d**) plots the difference in standard deviations of SCD as a function of time compared to a null distribution. Panel (**e**) indicates the mean bPL factor in both hypnosis and baseline conditions, and Panel (**f**) compares the difference in bPL between conditions to a null distribution. Null distributions in Panels (**c**), (**d**), and (**f**) were arrived at by calculating 1000 random permutations, shown individually in light grey lines.

Spectral power differences between the baseline and hypnosis conditions are present most notably at frequencies above the 24 Hz range. Higher frequencies are more pronounced in hypnosis, especially in the occipital region, while in the frontal area hypnosis is characterized by a decrease in the lower frequency range (see Fig. 1). This shows that spontaneous EEG activity markedly differs between the two conditions. The spatiotemporal dynamics of source-reconstructed, TMS-evoked activation under the baseline condition were in line with previous reports (Garcia *et al.* 2011); see Fig. 2a. TMS first elicited activation around the stimulated area, then spread to the parietal and frontal cortices. TMS during hypnosis first elicited activation in the posterior cortical and parietal areas, around 20 and 50 ms after the TMS pulse; see Fig. 2b. Subsequently (150–200 ms), the centroparietal activity elicited by TMS became strongly diminished compared to baseline. Importantly though, TMS-evoked activation persisted longer during hypnosis than baseline (250–300 ms). Although TMS evoked longer-lasting activation, it failed to evoke the strong, coherent activation in the frontoparietal circuits (around 150–200 ms) associated with conscious processing (Dehaene and Changeaux 2011; Koch *et al.* 2016). Differences in SCD between hypnosis and baseline conditions illustrate this difference when summed over all dipoles (Fig. 2c). As shown in Fig. 2d, there is more deviation in the source activations across trials in the hypnosis condition when compared to the baseline condition (cortical distribution of standard deviations is not presented as this effect did not localize to any specific cortical area). The temporal evolution of the bPL in the hypnosis condition is characterized by a fast first peak at 50 ms, followed by a general decrease compared to baseline. Baseline phase-locking was most prominent in the 100–200 ms timewindow (Fig. 2e and f).

**Figure 3.**
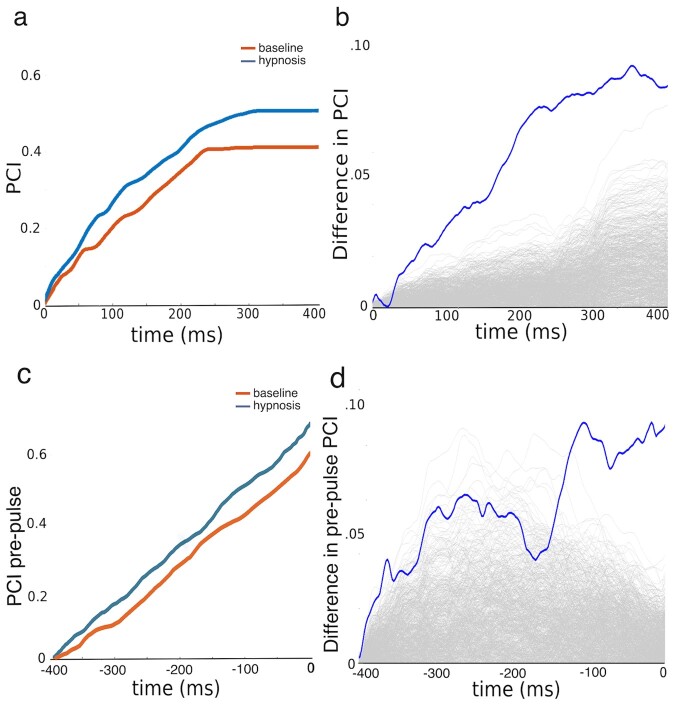
Differences in PCI during hypnosis and baseline. Panel (**a**) plots cumulative PCI as a function of time under baseline and hypnosis following TMS perturbation. (**b**) Observed difference in PCI between hypnosis and baseline compared to a null distribution. (**c**) Difference in cumulative PCI before the TMS perturbation. (**d**) Observed difference in pre-pulse PCI compared to a null distribution. Null distributions were arrived at by calculating 1000 random permutations, shown individually in light grey lines in Panels (**b**) and (**c**).

As shown in Fig. 3a, PCI remained within the normal variation observed in neurologically healthy individuals (Casarotto *et al.* 2016), but hypnosis was associated with more complex (more highly differentiated) activation patterns compared to baseline wakefulness: i.e. hypnosis significantly increased PCI throughout the TMS-evoked activation period; see Fig. 3b. To examine if similar effect could be detected from the spontaneous, not-TMS-evoked variation in EEG we ran similar PCI analysis but restricted it to the pre-TMS time window. As shown in Fig. 3c and d, similar but weakened effect was observed.

## Discussion

The results show that hypnosis can be accompanied by significant changes in brain state as measured both by PCI and TMS-evoked activation. To our knowledge, this is the first demonstration of increased PCI under a non-pathological conscious condition. An increase in magnetoencephalography signal complexity has previously been found using a related non-perturbational measure (Lempel-Ziv complexity) for the neural effects of DMT (Timmermann *et al.* 2019) ketamine, psilocybin, and LSD (Schartner *et al.* 2017). This change was accompanied by an increase in the power of higher frequencies under the hypnosis condition.

We propose that the observed change in brain state under hypnosis may be characterized by a shift from the metastable state of normal wakeful consciousness towards more segregated connectivity. We have demonstrated such shift in one participant, but further studies are required to examine how well it generalizes to hypnosis in general. In periods of normal consciousness, the neural activity in a set of cortical regions can transiently lock into a synchronized configuration (Dehaene and Changeaux 2011; Koch *et al.* 2016) but then flexibly reconfigure itself based on internal and external factors (Kelso 2012; Tognoli and Kelso 2014). In this study, the pattern of transient locking is revealed under baseline by strong and widespread activation in frontoparietal areas 150–200 ms after TMS pulse. In contrast, under hypnosis, this synchronized activity fails to initiate; processing in different cortical areas remains segregated, reflected in higher PCI values. In other words, in a normal waking state, the system can resolve the perturbation by integrating the pulse activity more effectively, whereas under hypnosis the coordination breaks down as the system is less integrated. Across trials, source activity showed more variation in the hypnosis condition already in the pre-pulse timewindow, consistent with the idea of a more segregated state under hypnosis. This difference is most pronounced following the TMS-pulse and again in the 150 ms time window. Differences in phase-locking were observed, strengthening the case for an alteration in the underlying metastable regime (Kitzbichler *et al.* 2009). Decreased phase-locking, as observed here in the 100–200 ms post-pulse timewindow in the hypnosis condition, is consistent with a decreased inter-area communication in the functional network (Modolo *et al.* 2020). Taken together, the PCI results and the decreased phase-locking form a consistent view of the brain, and occupying a more segregated state during hypnosis.

The shift from metastable to more segregated state could explain the behavioural and experiential changes accompanying hypnosis. Hypnotic states are behaviourally inflexible; they lack the normal spontaneous adaptive reactivity to internal or external stimuli because the brain’s specialized modules are not able to synchronize communication. Nevertheless, the capacity to react to suggestions is high, indicating a large variety of potential states: i.e. hypnotic suggestion can facilitate configurations not typically present during normal consciousness. Experientially, hypnosis is associated with a narrowed scope of conscious content; the information processed in specialized cortical regions is segregated from the global stream of consciousness (Dehaene and Changeaux 2011).

The analyses of pre- and post-pulse complexity suggest that while PCI calculated following perturbation allows for a faster and arguably more reliable way to assess complexity, the perturbation itself is not necessary for the calculation. There is, however, a higher signal-to-noise ratio in pre-pulse complexity as compared to post-pulse PCI.

Because we applied TMS to occipital cortex, one could argue that the observed difference between hypnosis and baseline conditions reflects the differences in phosphene perception. We did not inform the participant about the possibility of phosphenes before the experiment, because attending to phosphenes is known to increases the chances of perceiving them (Bestmann *et al.* 2007). After the experiment, the participant did not report perceiving phosphenes when queried.

## Considerations on the Single Subject Design

While the single-subject design poses problems for generalizability, we consider it to be well suited for this particular question. The history of neurosciences and psychology showcase several areas where single cases have proven invaluable for scientific understanding and advancement, an approach that has recently been suggested as an invaluable addition to the neuropsychology research design toolkit (Cubelli and Della Sala 2017). Crucial advances have been made possible by studying, e.g. cases such as Phineas Gage (Harlow 1848), patient H.M. (Scoville and Milner 1957), or Leborgne (Broca 1861). These studies have opened new hypotheses and theories for further investigation to be carried out in a broader sample with more variation. Here, we have demonstrated the changes in neural dynamics in what is undeniably an exemplar of a hypnotic individual. But we do not wish to imply that the current case would be a prototypical case of hypnosis. Quite the contrary, hypnosis is not necessarily a uniform phenomenon, but one that may include various different exemplars. Neutral hypnosis can—at least in this subject—substantially change the dynamical state of the brain, but it does not necessarily follow that similar alterations accompany every subject or hypnotic experience. This is a question for future research. Given that there is inter-individual variation in both normal waking PCI and suggestibility, a repeatable involuntary neural alteration within one person allows new considerations for empirical research in more varied settings.

Related to the debate on whether hypnosis occupies a distinct state of consciousness or lies on a socio-cognitive continuum, we can say that neutral hypnosis at the very least can cause a response in brain dynamics that clearly differs from the response in relaxed non-hypnotic brain configuration. Whether this qualifies as an altered state or not seems to be a merely semantics, and dependent on the exact way the contentious term ‘altered state’ is defined. Our results, which focus on neural state changes, obviously do not refute other approaches to hypnosis, such as the socio-cultural model. We see no discrepancy in hypnosis resulting in, both, an altered brain state, as well as being mediated by socio-cognitive factors or expectations.

Finally, we want to comment on why we did not include in this study a control group. In many studies on hypnotic participants, the results are compared to a group of participants who aim to ‘simulate’, e.g. the outcome of the hypnotic suggestion (e.g. Scheibe *et al.* 1968; Franz *et al.* 2020). We think this approach has merits, but it would not have worked in this study. First, what would have been the exact instruction for simulators? To relax, to concentrate, to meditate, to focus on a specific mental image, or just to generally ‘fake being hypnotized’? It seems that if we were to find a difference given a specific control condition one could always argue that it was not the ‘correct’ control condition. The control condition is easier to find in task-specific hypnosis. Second, the between-subjects comparison would be beside our point: What we wish to show is that a one-word hypnotic induction by itself can at least in one participant induce large scale changes in brain dynamics. Given that this participant has previously in well-documented research provided demonstrations in phenomena best characterized as hypnosis (including studies which employ control groups; see e.g. Kallio and Koivisto 2016), we feel confident in arguing that the phenomena we can here observe in the brain level is what is commonly termed as neutral hypnosis. For instance, a control group was unable to simulate changes in her volitional and involuntary eye movements (optokinetic reflex, the pupillary reflex, and programming a saccade to a single target) following a hypnotic induction (Kallio *et al.* 2011). Similarly, in a study by Kallio *et al.* (2017) TS-H was compared to a control group in a hypnotically induced altered colour perception task. She outperformed control group participants who were explicitly instructed to simulate the task using various cognitive strategies. This together with previous EEG findings of an arguably preconscious (70–120 ms after stimulus onset) ERP response following a suggested colour perception alteration (Koivisto *et al*. 2013) reduces the likelihood of her simulating the hypnotic responses, but of course such possibility cannot be ruled out in entirety. While people inhabiting other positions on the hypnotizibility continuum may or may not rely on similar changes in brain dynamics, here at least we have a person whose hypnotic responses seem contingent to such changes.

We acknowledge that the possibility that the participant was merely behaviourally faking hypnosis, and concurrently wilfully modulating her brain activity, cannot be ruled out. While PCI is arguably less susceptible to voluntary influences that more standard brain imaging approaches, the question of whether PCI can be modulated voluntarily (e.g. through meditation or via other cognitive manipulation) remains open. We welcome research into the effects of more nuanced manipulations—such as meditation, attention, and cognitive task load—on PCI, to better understand the implication of the present results. One should be mindful of the limitations of this single study on drawing too general explanations of hypnosis in general. Cumulative evidence from replications and varied research designs will allow for more certainty upon which to understand the brain changes accompanying hypnosis.

More broadly, we hope our results will spawn new research programmes and further theoretical advancements for the study of hypnosis. For example, the formulation of the possible mechanisms in terms of metastability alterations and point towards specific methodologies within functional magnetic resonance imaging, or towards magnetoencephalography for questions to do with more intricate dynamic behaviour. Theoretically, we could then move towards mechanisms, by, e.g. combining the way this state-specific change in the dynamics can moderate top-down predictions. Furthermore, a question remains of whether the segregation observed here is introduced gradually or whether it is introduced by a punctuated shift given a certain threshold. As such our research invites a future study on people occupying various positions on the suggestibility spectrum. Such a study would simultaneously function as a replication for this finding, and possibly resolve the problem of generalizability inherent in single-subject studies.

## Supplementary Data

Supplementary data are available at *NCONSC Journal* online.

## Supplementary Material

niab002_Supplementary_Data

## Footnotes

1 In (Casarotto *et al.*, 2016), Table 1, the median PCI was 0.53, ranging from 0.21 to 0.70 in 102 awake healthy controls.

2
https://www.nitrc.org/projects/cleanline (accessed 2019-03-03).
