# Supplementary material for: Segregated brain state during hypnosis
Source: Neurosci Conscious. 2021 Mar 10;2021(1):niab002. doi: 10.1093/nc/niab002 (PMC7959214; doi:10.1093/nc/niab002)
Supplement: niab002_Supplementary_Data [file niab002_supplementary_data.docx]

**SUPPLEMENTARY FIGURES**


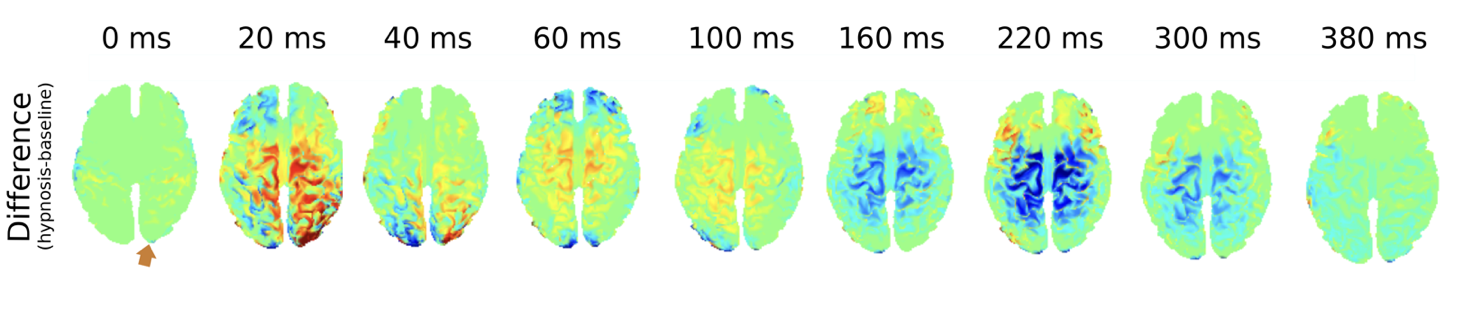


**Figure S1.** Differences in spatiotemporal source-current activations calculated by subtracting the significant sources in hypnosis from those of baseline. An orange arrow indicates the site of TMS stimulation.


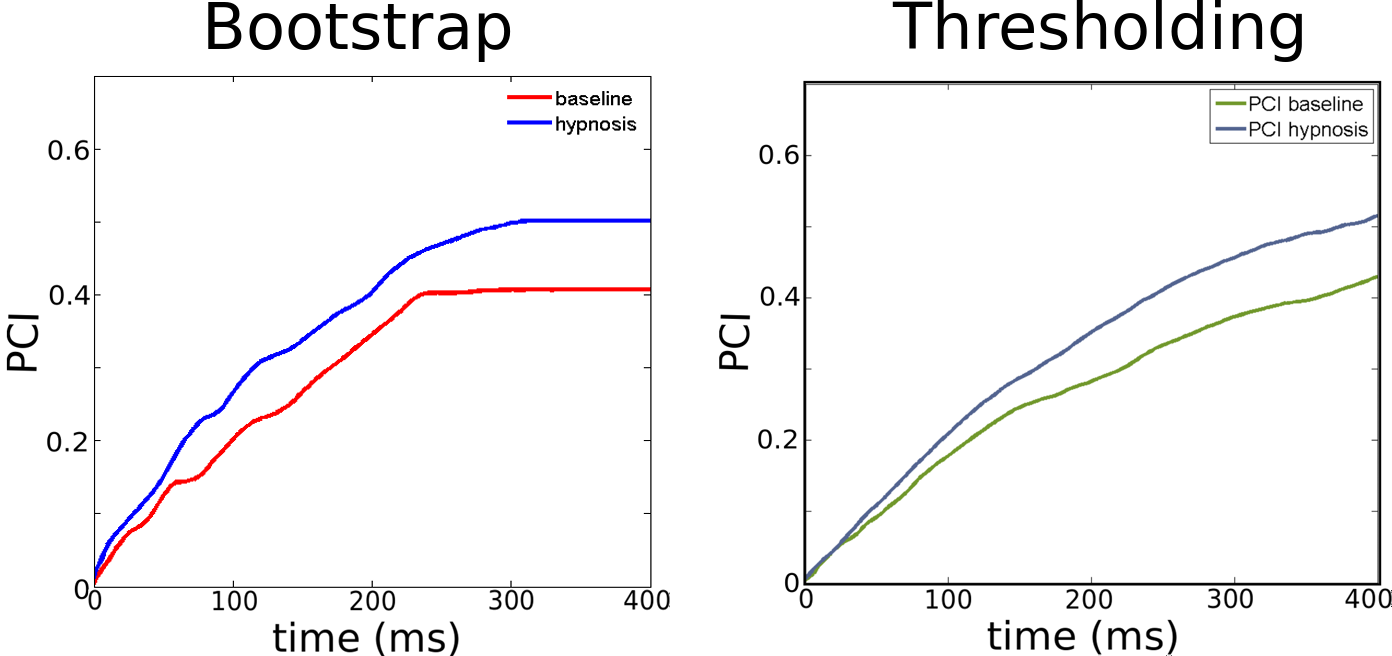


**Figure S2.** Comparison between two methods for calculating PCI. The *bootstrap procedure* is calculated from the mean of single trials, the *threshold procedure* from the mean of absolute values via a Hilbert transform. While some differences in the temporal evolution of PCI emerge, the overall result remains the same.
